# Human Immunodeficiency Virus gag and protease: partners in resistance

**DOI:** 10.1186/1742-4690-9-63

**Published:** 2012-08-06

**Authors:** Axel Fun, Annemarie MJ Wensing, Jens Verheyen, Monique Nijhuis

**Affiliations:** 1 Department of Virology, Medical Microbiology, University Medical Center Utrecht, HP G04.614, Heidelberglaan 100, Utrecht, 3584 CX, The Netherlands; 2 Institute of Virology, University of Cologne, Cologne, Germany

**Keywords:** HIV, Particle maturation, Protease inhibitors, Maturation inhibitors, Gag mutations, Resistance, Cross-resistance

## Abstract

Human Immunodeficiency Virus (HIV) maturation plays an essential role in the viral life cycle by enabling the generation of mature infectious virus particles through proteolytic processing of the viral Gag and GagPol precursor proteins. An impaired polyprotein processing results in the production of non-infectious virus particles. Consequently, particle maturation is an excellent drug target as exemplified by inhibitors specifically targeting the viral protease (protease inhibitors; PIs) and the experimental class of maturation inhibitors that target the precursor Gag and GagPol polyproteins. Considering the different target sites of the two drug classes, direct cross-resistance may seem unlikely. However, coevolution of protease and its substrate Gag during PI exposure has been observed both *in vivo* and *in vitro*. This review addresses in detail all mutations in Gag that are selected under PI pressure. We evaluate how polymorphisms and mutations in Gag affect PI therapy, an aspect of PI resistance that is currently not included in standard genotypic PI resistance testing. In addition, we consider the consequences of Gag mutations for the development and positioning of future maturation inhibitors.

## Review

### HIV maturation

**Figure 1:**
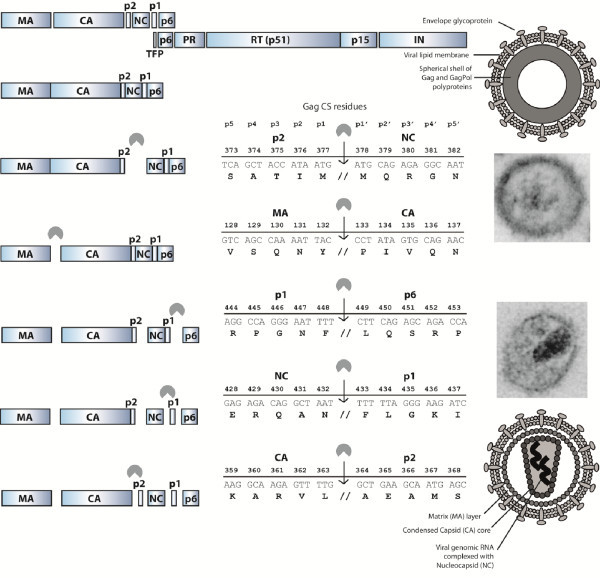
**A schematic representation of HIV particle maturation.** At the top, the viral GagPol polyprotein is depicted. On the left, the 5 sequential proteolytic processing steps of Gag are shown. In the middle, the 5 Gag cleavage sites (CS) and their nucleotide and corresponding amino acid sequences are shown. The numbers above the residues correspond to their position in the Gag polyprotein. At the top of the middle panel, the location of the p5-p5’ positions is indicated. On the right are schematic representations and electron-microscopy images of an HIV particle. Top: the immature, non-infectious particle with its granulated core. Bottom: the fully mature and infectious virion with its characteristic electron-dense conical core. The pacman figure represents the viral protease enzyme.

HIV is released from the host cell membrane as a non-infectious particle that is called the immature virion. After budding and release, the virion undergoes a dramatic structural rearrangement that results in fully infectious virus. Transition of the amorphous, non-infectious virion into the mature, infectious virion that is characterized by an electron-dense conical core is called maturation (Figure 1). This transition is triggered by the proteolytic cleavage of the Gag (Pr55^Gag^) and GagPol (Pr160^GagPol^) precursor polyproteins by the viral enzyme protease (PR). Gag is cleaved into the structural proteins matrix (MA, p17), capsid (CA, p24) and nucleocapsid (NC, p7), p6 and two small spacer peptides (p1 and p2). Pol, which is translated as the GagPol polyprotein after a -1 nucleotide frameshift event, that occurs with a frequency of 5-10% [1], encodes the viral enzymes PR, reverse transcriptase (RT) and integrase (IN). Analysis of different Gag substrates revealed that HIV PR recognizes the asymmetric, 3-dimensional conformation of the Gag substrate, rather than a particular peptide sequence [2]. The peptides that form the different cleavage sites (CS) have a superimposable secondary structure, yielding the so-called substrate envelope which fits within the substrate binding pocket of the viral PR. However, each substrate has a unique structure, and there are subtle differences in the way the amino acids protrude from the substrate envelope. It is thought that these small differences in substrate structure impact affinity for the viral protease and contribute to the highly regulated and ordered stepwise process of viral maturation in which all the individual cleavages occur at different rates [3-6] (Figure 1). First, the scissile bond between p2 and NC (MA-CA-p2↓NC-p1-p6) is cleaved, followed by separation of MA from CA-p2 (MA↓CA-p2). Subsequently p6 is cleaved from NC-p1 (NC-p1↓p6). Finally, the two small spacer peptides are removed in the rate-limiting cleavage steps NC↓p1 and CA↓p2, of which CA↓p2 is thought to be the final cleavage (Figure 1). This ordered cleavage is mainly regulated by those amino acids in the substrate that are in direct contact with the viral PR (p4-p3’ position, Figure 1) [6-8]. Although most studies have focused on the impact of these substrate residues that are in direct contact with the viral PR, it has been demonstrated that the more distantly located p4’ and p5’ residues can also affect processing efficiency [9-13].

### HIV protease

HIV protease is a member of the family of aspartic proteases and is a symmetrically assembled homodimer consisting of two identical subunits of 99 amino acids. Both subunits contribute catalytic residues to the active site (an aspartic acid at codon 25) [14,15]. The substrate binding pocket is at the center of the dimer and interacts with the different substrate sequences in the Gag and GagPol polyproteins. The mechanism that activates the viral PR, which is embedded in the GagPol polyprotein itself, is not yet fully understood. It is known however, that the viral PR is responsible for its own release from the precursor polyprotein (autoprocessing). Since PR is active only as a dimer, it is thought that autoprocessing is initiated by dimerization of two protease domains that are still embedded in the GagPol precursor. The initial cleavage is a transient, intramolecular event and the low occupancy of the embedded dimer configuration can explain its low enzymatic activity compared to the fully matured PR enzyme [16,17].

### Protease inhibitors

Detailed structural knowledge of HIV PR and its substrate led to the development of specific protease inhibitors (PIs). To date, nine different PIs have been approved for clinical use: saquinavir (SQV), ritonavir (RTV), indinavir (IDV), nelfinavir (NFV), (fos)amprenavir (FPV/APV), lopinavir (LPV), atazanavir (ATV), tipranavir (TPV) and darunavir (DRV). All PIs, with the exception of tipranavir, are competitive peptidomimetic inhibitors, mimicking the natural substrate of the viral PR. The peptidomimetic inhibitors contain a hydroxyethylene core which prohibits cleavage by the viral PR [18-25]. Instead of a hydroxyethylene core, tipranavir contains a dihydropyrone ring as a central scaffold [26]. In general, all these compounds have been designed to bind to the substrate binding region of the mature viral PR dimer with high affinity, but they tend to occupy more space than the natural substrate. For tipranavir and darunavir, it has been demonstrated that they have a dual mechanism of inhibition as they also impede dimerization of the viral PR [27]. This may contribute to their antiviral potency and high genetic barrier towards resistance, although the impact of the anti-dimerization activity has not been elucidated yet.

Inhibition of the initial GagPol processing steps which involve self-cleavage of the embedded HIV PR from the GagPol polyprotein (autoprocessing) would prevent viral maturation at the earliest stages and therefore be an ideal drug target. However, all PIs have been developed to bind the active site of the mature PR dimer, and it was shown that the embedded HIV PR dimer is 10,000 fold less sensitive to RTV than the mature PR dimer [16]. More recently, two different groups demonstrated independently and using different assays, that of the nine approved PIs, DRV and SQV are the most potent inhibitors of autoprocessing. However, both inhibitors are still three orders of magnitude less active against the embedded dimer compared to the mature PR dimer [28,29].

Currently, first line highly active antiretroviral therapy (HAART) regimens usually consist of a combination of two nucleoside reverse transcriptase inhibitors (NRTIs) with either a non-nucleoside reverse transcriptase inhibitor (NNRTI), an integrase inhibitor, or a PI. Effective HAART has reduced HIV-related morbidity and mortality and greatly improved therapeutic success rates [30]. However, in the early days of PI therapy, high pill burden and related toxicity, low bioavailability and a low barrier to the emergence of resistance severely impaired effective treatment of HIV infected individuals. Resistance to PIs was usually associated with the selection of multiple mutations in the viral PR resulting in broad class cross-resistance. Since then, several strategies have been developed to improve clinical outcome and increase the barrier to development of PI resistance. Co-administration with ritonavir, an inhibitor of the cytochrome P450 3A4 isoenzyme, which is involved in the metabolism of all PIs, considerably improved the bioavailability and half-life of PIs [31] resulting in higher PI plasma concentrations with a reduced pill burden and related drug toxicity [32]. Another improvement was the development of second generation PIs that have an intrinsic higher genetic barrier to development of resistance, which are (fos)amprenavir, lopinavir, atazanavir, tipranavir and darunavir [20,23-26,33-36]. In patients on second generation PI based HAART and who have not received prior PI therapy, selection of primary resistance mutations in the viral PR is rare, even in case of therapy failure.

Evolution of PI resistance usually has a biphasic signature in which mutations develop initially in or near the substrate binding pocket of the viral PR. In fact, it has been shown that resistance mutations mainly develop where the PIs protrude beyond the substrate binding pocket at residues that are in direct contact with the inhibitor, but not with the natural substrate [37-39]. These mutations lower the affinity for the drug more than for the natural substrate, which decreases the susceptibility to the drug, resulting in a resistant virus. However, by changing the substrate binding region of the enzyme, the affinity for the natural substrate (Gag) is also slightly altered, often resulting in reduced viral replication [40-42]. In a second step, compensatory or secondary mutations can be selected that restore viral replication and/or enhance drug resistance. These mutations are found in the viral PR itself as well as in the Gag substrate and in particular, in the NC/p1 and p1/p6 cleavage sites [9,12,43-49]. It has also been shown that several Gag substrate mutations are primary drug resistance mutations that confer PI resistance in the absence of PR mutations [10,50].

In this review we describe the results of a comprehensive search of the available literature investigating natural variation in Gag and coevolution of Gag and protease during protease inhibitor exposure. We provide a detailed overview of Gag mutations that are observed during PI exposure, both *in vivo* and *in vitro* and how they affect PI therapy and resistance. Furthermore, we evaluated the impact of these Gag mutations on the efficacy of the novel antiretroviral class of CA/p2 maturation inhibitors.

### Natural variation of Gag cleavage sites

Only a limited number of studies evaluated the natural variation within Gag and its cleavage sites and most data are from studies focusing on subtype B [51-58]. The limited data that are available suggest that the variation in non-B subtypes is greater than in subtype B [52,53,56]. All these studies show that the degree of conservation differs dramatically between individual amino acid positions as well as between the different cleavage sites as a whole (Table 1). Cleavage site p2/NC is the most variable of the 5 Gag cleavage sites, followed by p1/p6, NC/p1, CA/p2 and finally MA/CA, which is the most conserved CS in subtype B isolates. Amino acids 369-371 in p2 are included in this table as they are important for CA/p2 maturation inhibitor susceptibility, which will be described later in this review.

**Table 1:** Natural variation of Gag cleavage sites in subtype B isolates

			**MA**			//			**CA**					
HXB2 aa	V	S	Q	N	Y	//	P	I	V	Q	N			
position	128	129	130	131	132	//	133	134	135	136	137			
variability (%)	3.5	-	4.3	-	3.5	//	-	-	0.9	0.9	-			
			**CA**			//			**p2**					
HXB2 aa	K	A	R	V	L	//	A	E	A	M	A	Q	V	T
position	359	360	361	362	363	//	364	365	366	367	368	369	370	371
variability (%)	0.2	0.2	1.8	11.5	1.2	//	-	-	-	-	1.2	4.1	24.8	16.4
			**p2**			//			**NC**					
HXB2 aa	S	A	T	I	M	//	M	Q	R	G	N			
position	373	374	375	376	377	//	378	379	380	381	382			
variability (%)	36.3	32.6	42.7	23.6	1.8	//	5.5	-	40.9	5.5	2.1			
			**NC**			//			**p1**					
HXB2 aa	E	R	Q	A	N	//	F	L	G	K	I			
position	428	429	430	431	432	//	433	434	435	436	437			
variability (%)	2.3	3.5	-	0.5	-	//	-	0.1	-	6.3	5.5			
			**p1**			//			**p6**					
HXB2 aa	R	P	G	N	F	//	L	Q	S	R	P			
position	444	445	446	447	448	//	449	450	451	452	453			
variability (%)	-	0.1	-	-	-	//	9.1	-	22.8	-	8.4			

Data describing the natural variation at individual codons in the five Gag cleavage sites were pooled and the frequency of polymorphisms as compared to the HXB2 reference sequence was calculated. Only data describing subtype B isolates were included in the analysis. Codons 369-371 in p2 are included because of their important role in CA/p2 maturation inhibitor susceptibility.A dash indicates 100% conservation.

### Selection of Gag cleavage site mutations during protease inhibitor exposure

During PI exposure, substitutions in all Gag CS have been described. Mutations in MA/CA (codon 128), NC/p1 (codons 431, 436 and 437) and p1/p6 (codons 449, 452 and 453) are observed most frequently *in vitro* and *in vivo* and have been shown to confer PI resistance (Table 2). The effect of these different CS mutations is described in detail below.

**Table 2:** All Gag mutations associated with PI exposure and/or resistance and maturation inhibitor resistance

**Gag mutation**	**Associated with PI exposure**		**Associated with PI resistance**	**Associated with maturation inhibitor resistance**
	***in vivo***	***in vitro***		
**MA**				
E12K		yes [59,60]		
G62R		yes [61]		
L75R		yes [59,60]		
R76K	yes [62,63]		yes [62]	
Y79F	yes [62,63]		yes [62]	
T81A	yes [62,63]		yes [62]	
K112E		yes [64]		
**CS MA/CA**				
V128I/T/A/del	yes [56,65,66]	yes [64]	yes [64]	
Y132F	yes [46,67]			
**CA**				
M200I		yes [64]		
H219Q/P		yes [59,60,64]		
**CS CA/p2**				
A360V	yes [46]			
V362I		yes [68]		yes [69,70]
L363M/F/C/N/Y		yes [61]		yes [69,71-74]
S368C/N		yes [51,54]		yes [69]
**p2**				
Q369H	yes [54]			yes [72,74]
V370A/M/del	yes [54]			yes [69,70,72,74-76]
T371del	yes [77]			yes [72,74,76]
**CS p2/NC**				
S373P/Q/T	yes [51,54,78]			
A374P/S	yes [78]			
T375N/S	yes [46,78]			
I376V	yes [46,51]			
G381S	yes [46]			
**NC**				
I389T	yes [77]			
V390A/D		yes [59,60]		
I401T/V	yes [77]	yes [64]		
R409K		yes [59-61,64]		
**CS NC/p1**				
E428G	yes [66]			
Q430R		yes [44]		
A431V	yes [10,45,46,51,55,57,77-83]	yes [10,44,84,85]	yes [13,55,81]	
K436E/R	yes [10,55,82]	yes [10]	yes [10,13,55]	
I437T/V	yes [10,45,46,51,55,78,82,86]	yes [10,61]	yes [10,13,55]	
**CS p1/p6-gag**				
L449F/P/V	yes [9,45,46,55-57,67,77,79,80,82,86]	yes [44,49,85,87]	yes [55]	
S451T/G/R	yes [55,66]			
R452S/K	yes [55,66,79,82]	yes [87]	yes [55]	
P453A/L/T	yes [9,55,57,77,81,82][67]	yes [84,87]	yes [9,55]	
**p6-gag**				
E468K		yes [59]		
Q474L	yes [77]			
A487S	yes [77]			
P497L	yes [77]			
**p6-pol**				
V484G/I/P/S	yes [88]			
**CS TFP/p6pol**				
D437N	yes [56,64]			

#### MA/CA mutations

Several mutations at MA codon 128 (V128T/A/del) were associated with exposure to PIs *in vivo* (FPV/ATV/r) [56]. In addition, substitution V128I was observed more frequently in subtype G isolates from PI experienced patients compared to PI naïve patients [65]. Mutation V128I was also associated with virological rebound in patients on a boosted DRV containing regimen and was positively correlated with presence of PR mutation V32I [66]. It also has been selected *in vitro* with GS-8374, an experimental high genetic barrier PI [64].

#### NC/p1 mutations

NC/p1 CS mutation A431V is the most frequently occurring Gag CS mutation during PI exposure. It has been observed *in vivo* during PI therapy with RTV [46,51,79,80], IDV [45,51], NFV [77], SQV [51,79], LPV [81] and was also associated with PI exposure in unspecified therapy or cross-sectional analyses [10,55,57,78,82]. It is often observed in combination with one or more of the following PI resistance mutations in the viral PR: L24I, M46I/L, I50L, L76V, V82A/T/F and I84V. *In vitro*, mutation A431V was selected during exposure to RTV [10,84], LPV [85] and experimental PI BILA 2185 BS [44]. Mutation A431V confers resistance to all PIs except DRV [10,55] and can be considered a primary PI resistance mutation as it confers PI resistance in the absence of mutations in the viral PR [10]. The level of resistance caused by this mutation is comparable to that of single PI resistance mutations in PR (M46I and V82A) [81].

Substitutions at amino acids 436 and 437 in the NC/p1 CS have been observed during PI therapy with RTV [46,51], IDV [45], SQV [51] and were associated with PI exposure in unspecified therapy or cross-sectional analyses [10,55,78,82,86]. Mutations at Gag position 436 are associated with PR mutation V82A and mutation I437V with PR mutations: I54V, V82F/T/S and I84V. They have been selected *in vitro* with experimental high genetic barrier PIs (RO033-4649; 436E + 437T, 437 T and 437V [10] and (GRL-02031; 437T [61]). These mutations confer PI resistance and mutation I437V and the double mutation K436R + I437T also confer PI resistance in the absence of PR mutations [10,13,55]. Mutation I437V alone results in low-level PI resistance, but the double mutation K436E + I437T has a greater impact on PI susceptibility and confers slightly more resistance than mutation A431V [13].

#### p1/p6 mutations

Mutations in the p1/p6 CS and especially substitutions at codons 449, 452 and 453 are also often observed during PI therapy [9,45,46,55-57,77,79-82,86].

Mutations L449F/V/P have been associated with PI therapy in a number of cross-sectional studies (Table 2) and have been directly related to treatment with RTV [80], IDV [45],NFV [67], FPV, ATV [56], SQV [46,56] and APV [9]. Mutation L449F often occurs in combination with PR mutations D30N/N88D, I50V and I84V and mutation L449V is observed with PR mutations I54L/M/S/TA.

L449F has been selected *in vitro* using LPV [85], APV [49], and experimental PIs BILA 1906 BS, BILA 2185 BS [44] and GW640385 [87]. Alone, mutation L449F has no effect on PI susceptibility, but in combination with mutations in the viral PR it affects inhibitor resistance. Combined with D30N/N88D, it decreases susceptibility to IDV, SQV, APV and TPV. In combination with V82A or V82A/L90M, mutation L449F decreases susceptibility to all PIs (DRV was not tested). Interestingly, when combined with PR mutation I50V, it induces hypersusceptibility to IDV, LPV and especially RTV [87].

Amino acid substitutions at position 452 have been associated with exposure to RTV, SQV [79], DRV [66] and in two cross-sectional studies [55,82]. *In vivo*, mutations at this position associate with PR mutations D30N/N88D, I50V and I84V [55]. *In vitro*, mutation P452K has been selected with experimental PI GW640385 [87]. In combination with PR substitutions I84V or I84V/L90M, R452 mutations decrease susceptibility to all PIs except TPV (DRV was not tested) [55].

Mutations at position 453 have been associated with PI exposure *in vivo* to APV [9], LPV [81], NFV [57,67], RTV, IDV and SQV [57]. It is often seen together with PR mutations D30N, I50V, I84V, N88D and L90M [9,55,57]. *In vitro*, mutation P453L has only been described being selected with IDV and P453T with experimental PI GW640385 [87]. P453L does not confer resistance on its own, but enhances PI resistance in combination with PR mutation I50V [9,55] and I84V to NFV, APV and SQV and to all PIs in combination with L90M (DRV not tested) [55].

#### Gag CS mediated PI resistance

The observed association between PI exposure and Gag CS mutations signifies the close relationship of the viral PR and the Gag CSs and their contribution to escape PI pressure. The effect of Gag CS mutations on PI susceptibility has been studied in detail. Structural and functional analyses of the processing efficiencies of wild type or mutant substrates showed improved processing and/or higher predicted binding affinities for the mutant substrates [9,11,37,89,90]. Enhanced processing of Gag is believed to shift the equilibrium of protease inhibitor-Gag substrate with the viral PR in favour of the Gag substrate and thereby confers resistance. This proposed mechanism of Gag CS mediated PI resistance is supported by studies that show altering just one CS (NC/p1 in these studies) also affects the processing efficiency at other CSs and thereby the entire substrate processing cascade [13,91]. Nevertheless, the difference in affinity of the viral PR for either the substrate or the PIs seems to contradict this explanation. Whereas the PR’s affinity constant for the PIs is thought to be in the low nanomolar range, the affinity constant for the natural substrates is in the millimolar range [92]. How a small shift in equilibrium induced by CS mutations can negate this million fold difference in binding affinity is not fully understood. One possible explanation that is offered comes from the stoichiometry of viral PR relative to its substrate, as it is present at the site of assembly and maturation [93]. The ratio between PR and natural substrate compared to PR and PI might be very different at the site of HIV maturation than in the cell-free environment used for *in vitro* enzymatic analysis which could strongly affect the actual *in vivo* kinetics [12].

### Selection of Gag non-cleavage site mutations during protease inhibitor exposure

Besides HIV-1 CS mutations, accumulation of non-CS mutations during PI therapy has been observed in all Gag proteins (MA, CA, NC, p6) as well as in spacer peptide p2 (Table 2). Additionally, several non-CS mutations in Gag have been identified *in vitro* to contribute to PI resistance in the presence of PR mutations [34,59,64], but can also mediate reduced PI susceptibility in the absence of PR mutations [62,94]. The impact of single non-CS Gag mutations on PI susceptibility has not been investigated, but combinations of non-CS Gag substitutions have been shown to affect both viral replication capacity and PI susceptibility [59,60,62,77]. The underlying mechanism of non-CS mediated resistance is not well understood. Since Gag non-CS mutations did not accumulate in functional related regions but were found throughout the whole Gag precursor protein, multiple mechanisms are likely to be involved and different per gene-segment.

#### Matrix

Substitutions in MA have been associated with virological failure against boosted LPV containing therapy (R76K, Y79F and T81A) [62,63]. They have been selected *in vitro* with APV (E12K and L75R [59,60]) and experimental PIs GRL-02031(G62R [61]) and GS-8374 (K112E) [64]). The mechanism of MA-mediated resistance remains elusive, but it is speculated that MA mutations change the multimerization of viral Gag. Another mechanism was suggested in a study demonstrating that changes in the tertiary protein structure of the Gag precursor proteins, caused by three mutations in the matrix protein, conferred drug resistance [62]. The three resistance associated residues (R76K, Y79F and T81A) are located in an alpha helical structure within MA, and the mutations result in loss of certain hydrogen bonds and thus more flexibility around the helix. It is hypothesized that the greater flexibility increases either the affinity or the availability/accessibility of the MA-CA CS with respect to the PR.

#### Capsid

Mutations in CA have not been associated with virological failure to PI therapy, but substitutions M200I (with GS-8374 [64]) and H219Q/P (with GS-8374 [64] and APV [59,60]) have been selected during PI exposure *in vitro*. The effect of mutation M200I is unknown, but substitutions at codon H219 interfere with binding of cyclophilin A and possibly reduce the requirement for cyclophilin A for efficient replication and thereby increase viral replication [59].

#### p2

Mutations in p2 at codons 369-371 appear to accumulate during PI therapy [54,77], but they have not been associated with virological failure to PI therapy. They have also not been observed during *in vitro* selections with PIs or have demonstrated to contribute to PI resistance. The p2 mutations that accumulate during PI therapy are located in a proposed alpha helical structure spanning the CA/p2 CS [54]. Therefore, the selection by PIs of these mutations might be explained by changes in the stability or the conformation of the alpha helical structure influencing the accessibility of the CA/p2 CS by the viral PR.

#### NC

Amino acid substitutions in NC have been associated with NFV failure (I389T and I401V [77]). Other mutations in NC have been selected *in vitro* with APV (V390A/D and R409K [59,60]) and GS-8374(I401T and R409K [64]). None of these mutations has been demonstrated to confer PI resistance, and their contribution to PI therapy failure and PI resistance remains unclear.

#### p1

Mutations in p1 outside its cleavage sites have not been described to be associated to PI exposure and resistance, *in vivo* or *in vitro*.

#### p6-gag

Only a handful of mutations in p6 have been described in relation to PI exposure *in vivo* (NFV; Q474L, A487S and P497L [77]) and *in vitro* (APV; E468K [59]). E468K in combination with other mutations improves viral replication in the presence of PIs, but the mechanism has not been elucidated [59].

One paper describes (partial) duplication of the P(S/T)APP motif in relation to PI therapy response. P(S/T)APP is a proline rich domain in p6-gag (Gag aa 455-459) that recruits Tsg101, a cellular factor involved in HIV budding. The authors found a significant association of partial or complete P(S/T)APP duplication with a decrease in virological response to APV at week 12 in highly pre-treated but APV naïve patients [95]. In addition, P(S/T)APP duplications were significantly associated with the presence of a mutation at V82 in PR. The hypothesized mechanism is an increase in viral packaging efficiency and budding, leading to an enhanced viral fitness. One other study describes an accumulation of P(S/T)APP insertions/duplications during HAART, but it does not comment on the type of antiretroviral therapy [96]. In contrast, three other studies did not observe a correlation between P(S/T)APP duplications and antiretroviral therapy [97-99], and one study even found a non-significant trend that HIV-1 patients harbouring P(S/T)APP insertions were less likely to experience virological failure [100].

### Impact of Gag mutations on PI therapy

Only a few studies investigated the impact of Gag mutations on viral response during subsequent PI therapy. We summarize the available data of CS substitutions at MA codon 128, substitutions in p2/NC (codon 373), NC/p1 (codons 428, 431 and 437), p1/p6 (codons, 449, 451, 452 and 453) and non-CS p6Gag substitutions at codon 484.

Mutations at MA/CA CS 128 (3.5% natural variability in subtype B isolates (Table 1)) were negatively associated with virological response in ANRS 127, a trial involving naïve patients receiving one of two dual-boosted PI combinations (FPV/ATV/r or SQV/ATV/r) [56]. Mutation V128I was also observed in >10% of virological rebounders in an analysis of the combined POWER 1, 2 and 3 trials that evaluated virological response to DRV/r plus optimized background therapy in PI-experienced patients [66].

Mutation S373Q (codon 373 is highly polymorphic, 36% variability in subtype B isolates (Table 1)) in the p2/NC CS, which was associated with the emergence of specific PR mutations during SQV therapy (K20R/I/M and L89M/I) did not have an effect on the virological response. In contrast, mutation S373P negatively impacted virological response to SQV [78].

The frequently observed NC/p1 CS mutation A431V (highly conserved, 0.5% variability in subtype B isolates (Table 1)) was not associated with a poorer virological outcome in several studies [10,78] and remarkably, was correlated with a better outcome to DRV/r therapy [101].

Analysis of the NARVAL trial [102] revealed that mutation I437V (5.5% natural variability in subtype B isolates (Table 1)) was significantly associated with a reduced virological response to different PI therapies (RTV, IDV, NFV, SQV and APV) [10]. It was also associated with virological failure in patients on DRV containing therapy in absence of multiple primary DRV resistance mutations in the viral PR [103]. Conversely, mutations at this position in the pol open reading frame (CS TFP/p6pol) positively impacted virological response to double boosted PI therapy (FPV/ATV/r or SQV/ATV/r) [56].

The study on ANRS 127 also revealed a negative association of CS p1/p6 mutation L449P (9.1% natural variability in subtype B isolates (Table 1)) on virological response to (FPV/ATV/r and SQV/ATV/r) [56]. In addition, mutations E428G, S451T and R452S (2.3, 22.8 and 0% natural variability in subtype B isolates (Table 1)) were linked with a reduced response to DRV/r in the POWER trials [66]. In contrast mutations S451G/N/R were associated to a better virological outcome in patients receiving first-line LPV/r monotherapy [88]. This study also showed a negative effect on virological response to LPV/r monotherapy of non-CS Gag mutations at codon 484 (V484G/I/P/S).

Mutations at codon 453 (8.4% natural variability in subtype B isolates (Table 1)) were not associated with virological response [57,78].

### Maturation inhibitors

Similar to PIs, the novel class of maturation inhibitors prevents viral replication by inhibiting particle maturation, but instead of targeting the viral PR, they target the Gag and GagPol precursor proteins directly. Within this experimental class of antiretrovirals, the CA assembly inhibitors and CA/p2 inhibitors are the most advanced in their development. CA assembly inhibitors are thought to bind CA and inhibit particle maturation by interfering with CA-CA interactions required for the formation of the conical-shaped capsid core. The CA subunits consist of two domains, the N-terminal and C-terminal domains (NTD and CTD). The mature capsid is constructed as a lattice of hexamers and NTD-NTD, NTD-CTD as well as CTD-CTD interactions are required to build the hexamer lattice [104,105]. Binding of the CA assembly inhibitor disrupts the molecular interface between the functional N-terminal and C-terminal structures of CA, which are located adjacently in the hexamer lattice, thereby preventing core assembly. Both small molecules and peptide derivatives are being investigated as potential CA assembly inhibitors and examples of CA assembly inhibitors that were or are in development are: CAP-1 [106,107], CAI [108], NYAD-I [109], BI-257, BI-627 and BI-720 from Boehringer-Ingelheim [110], PF3450074 from Pfizer [111,112] and CAC1, CAC1M and H8 [113].

As the name suggests, CA/p2 inhibitors impede particle maturation by specifically blocking the cleavage of CA from p2, which is one of the final and rate-limiting steps in the Gag processing cascade (Figure 1). Unprocessed CA/p2 (p25) interferes with core assembly and results in the formation of non-infectious particles [91,114]. Most data on CA/p2 inhibitors are derived from work on bevirimat (BVM, Panacos PA-457, Myriad MPC-4326), which was the most advanced maturation inhibitor in its development (phase II clinical trials). Western blotting and *in vitro* resistance selection studies identified CA/p2 as the target region of bevirimat [71,114], which was later confirmed by cross-linking studies [115]. It has also been shown that bevirimat has a stabilizing effect on the immature Gag lattice which indicates that bevirimat already binds during assembly and must be incorporated to inhibit maturation [116]. This observation offers an explanation why CA/p2 inhibitors are unable to inhibit the processing of monomeric Gag in solution. CA/p2 inhibitors include bevirimat, PA1050040 which is a second generation maturation inhibitor from Panacos [117] based on bevirimat, two maturation inhibitors from Myriad Pharmaceuticals, Vivecon (MPC-9055) [118,119] and MPI-461359 [120], and PF-46396 [121] from Pfizer.

The initial *in vitro* selection studies with bevirimat identified resistance mutations in the CA/p2 CS at Gag positions 358, 363, 364 and 366 [71]. A more recent study identified additional resistance mutations at Gag codons 362, 368 and 370 [69]. Phase 2b clinical studies demonstrated that baseline polymorphisms (substitution and/or deletions) slightly downstream of the CA/p2 cleavage site (Gag p2 aa 369, 370 and 371, known as the QVT-motif) also confer resistance [72,122]. All currently known bevirimat resistance mutations are located in or near the CA/p2 CS (Gag 359-368) (Table 2[69-72,122,123]). The genetic barrier of CA/p2 maturation inhibitors appears to be low. Most single resistance mutations confer high levels of resistance. This is confirmed by the *in vitro* studies where, in contrast to protease and integrase inhibitors, during *in vitro* selections with bevirimat, no accumulation of mutations is observed.

### Impact of Gag mutations on CA/p2 maturation inhibitor susceptibility

Several amino acid positions in Gag that are known to affect CA/p2 maturation inhibitor susceptibility are highly polymorphic, including codons 362, 370 and 371 (Table 1). In the treatment-naïve population, approximately 30% of patients infected with subtype B harboured an isolate with at least one mutation associated with a reduced susceptibility to bevirimat [54], and this appears to be much higher in non-B subtypes with a prevalence from over 70% to as high as 93% [124,125].

Although PIs and maturation inhibitors have a different target site, this review clearly indicates that PI exposure can result in selection of mutations in Gag, including the CA/p2 cleavage site which affects CA/p2 maturation inhibitor susceptibility (Table 2). Several studies showed an accumulation of bevirimat resistance mutations during PI treatment in bevirimat naïve patients [54,124,126]. These mutations were mainly observed in the QVT-motif. In subtype B isolates with PI resistance, the prevalence of bevirimat resistance mutations increased to 45%, a statistically significant increase. Accumulation of mutations at 4 individual positions in the CA/p2 region was also statistically significant and involved amino acid substitutions S368C, Q369H, V370A and S373P (Table 2[54]). In addition, mutations associated with bevirimat resistance were significantly more detected in HIV isolates with ≥3 PI resistance mutations than in those with less than three PI mutations [54,124].

The data presented in this review show that the CA/p2 region is variable and affected by PI exposure. A reduced activity of maturation inhibitor activity can be expected in one-third of the treatment-naïve HIV-1 subtype B isolates and significantly more in PI resistant HIV. Moreover, one could speculate that even in those individuals who do not have mutations in the CA/p2 region, mutations in the viral PR may affect subsequent resistance development to the CA/p2 maturation inhibitor. One study demonstrated that an impaired Gag processing efficiency caused by PI resistance mutations, delayed the development of bevirimat resistance and reduced the level of bevirimat resistance conferred by bevirimat resistance mutations [127]. Conversely, we showed that an increased Gag processing efficiency can result in enhanced levels of bevirimat resistance [69]. Mutations at codons 362 and 368 give rise to low level bevirimat resistance (2-6 fold) in the presence of wild type PR, whereas in the presence of a drug resistant HIV PR with increased Gag processing bevirimat resistance increases to >150 fold [69]. This intricate relation between HIV PR and Gag cleavage is supported by a study by Doyon *et al.*, who also demonstrated that a CA/p2 mutation at position 362 has differential effects on CA/p2 processing depending on the genotype of the protease present in the virus [68].

## Conclusions

In conclusion, these studies indicate that during PI exposure, mutations in the target region of the CA/p2 inhibitors may be selected, reducing the baseline susceptibility to the maturation inhibitor. Furthermore, the level of Gag processing of a PI resistant isolate may impact the development of bevirimat resistance.

### Clinical perspective

This review highlights the complex interactions between the viral protease and its Gag substrates and how mutations in Gag can affect PI and maturation inhibitor susceptibility. The data summarized in this review clearly show that mutations in Gag accumulate during PI therapy and that these mutations can contribute to PI susceptibility. Even though contemporary therapy success rates are very high and development of primary resistance to PI containing HAART is rare, the relative high incidence of unexplained failure without major PI resistance mutations in PR supports including Gag in the resistance analysis.

Especially, the addition of the C-terminal region of Gag (NC/p1 and p1/p6gag CSs) to routine testing could substantially improve our knowledge on genetic variability and the predictive value of genotypic resistance testing. However, this coins a paradox as the actual contribution of Gag mutations to virological failure is largely unknown, and this question can only be answered by including Gag in genotypic testing in clinical trials and cohorts.

HIV maturation inhibitors target the Gag proteins directly, and therefore genotypic analysis of Gag is invaluable for the development and clinical implementation of these inhibitors. This review illustrates that naturally occurring Gag polymorphisms dramatically affect the susceptibility to maturation inhibitors in clinical studies. Furthermore, accumulation of mutations at these polymorphic positions in Gag is observed during PI therapy failure, strongly affecting the sequential utilization of maturation inhibitors. New and more potent maturation inhibitors should therefore overcome the resistance associated with these highly variable positions in Gag and exhibit synergy with protease inhibitors. They should capitalize on the reduced processing often caused by PI resistance mutations in such a way that there is added value from the use of a maturation inhibitor in salvage therapy for PI experienced patients.

## Abbreviations

HIV, Human Immunodeficiency Virus; PR, Protease; MA, Matrix; CA, Capsid; NC, Nucleocapsid; RT, Reverse transcriptase; IN, Integrase; CS, Cleavage sites; PI, Protease inhibitor; HAART, Highly active antiretroviral therapy; NRTI, Nucleoside reverse transcriptase inhibitor; NNRTI, Non-nucleoside reverse transcriptase inhibitor; NTD, N-terminal domain; CTD, C-terminal Domain; BVM, Bevirimat.

## Competing interests

The author declare that they have no competing interest.

## Authors’ contributions

AF and MN wrote the manuscript. AMJW modified and contributed parts of the manuscript in her role as clinical virologist. JV reviewed the literature and contributed parts of the manuscript. All authors read and approved the final manuscript.
